# Hollow mesoporous organosilica nanoparticles reduced graphene oxide based nanosystem for multimodal image‐guided photothermal/photodynamic/chemo combinational therapy triggered by near‐infrared

**DOI:** 10.1111/cpr.13443

**Published:** 2023-03-20

**Authors:** Chenguang Zhang, Yuting Cai, Dang Pengrui, Jiechen Wang, Lu Wang, Jiayun Xu, Yuhan Wu, Wenwen Liu, Lili Chen, Zhengtang Luo, Feilong Deng

**Affiliations:** ^1^ Hospital of Stomatology, Sun Yat‐sen University, Guangdong Provincial Key Laboratory of Stomatology Guangzhou China; ^2^ Guanghua School of Stomatology, Sun Yat‐sen University Guangzhou China; ^3^ Department of Chemical and Biological Engineering Hong Kong University of Science and Technology Hong Kong China; ^4^ Liaoning Provincial Key Laboratory of Oral Diseases, School and Hospital of Stomatology China Medical University Shenyang China; ^5^ Department of Stomatology Union Hospital, Tongji Medical College, Huazhong University of Science and Technology Wuhan China; ^6^ School of Stomatology, Tongji Medical College, Huazhong University of Science and Technology Wuhan China; ^7^ Hubei Province Key Laboratory of Oral and Maxillofacial Development and Regeneration Wuhan China; ^8^ Department of Geriatric Dentistry Peking University School and Hospital of Stomatology Beijing China

## Abstract

Developing a nanosystem that can perform multimodal imaging‐guided combination therapy is highly desirable but challenging. In this study, we introduced multifunctional nanoparticles (NPs) consisting of graphene oxide‐grafted hollow mesoporous organosilica loaded with the drug doxorubicin (DOX) and photosensitizers tetraphenylporphyrin (TPP). These NPs were encapsulated by thermosensitive liposomes that release their contents once the temperature exceeds a certain threshold. Metal oxide NPs grown on the graphene oxide (GO) surface served multiple roles, including enhancing photothermal efficiency, acting as contrast agents to improve magnetic resonance imaging, increasing the sensitivity and specificity of photoacoustic imaging, and catalysing hydrogen peroxide for the generation of reactive oxygen species (ROS). When locally injected, the HMONs‐rNGO@Fe_3_O_4_/MnOx@FA/DOX/TPP NPs effectively enriched in subcutaneous Hela cell tumour of mice. The photothermal/photodynamic/chemo combination therapy triggered by near‐infrared (NIR) successfully suppressed the tumour without noticeable side effects. This study presented a unique approach to develop multimodal imaging‐guided combination therapy for cancer.

## Graphical abstract

HMONs‐rNGO@Fe3O4/MnOx@FA/DOX/TPP nanoparticles for Fluorescence/Ma;gnetic/photoacoustic image‐guided photothermal/photodynamic/chemo combination therapy triggered by a single laser.

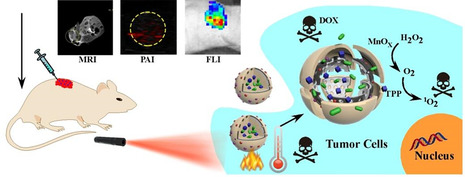

## 1 INTRODUCTION

The incidence rate of cancer is increasing yearly, making it one of the significant causes of death worldwide.^1^ While numerous therapeutic strategies have been developed, the complexity and specificity of the tumour microenvironment continue to present a challenge for effective tumour therapy.^2^ Imaging‐guided therapy, which can monitor tumour size in real‐time and provide controllable treatment under imaging guidance, could be a more efficient strategy for achieving precision therapy of tumours.^3^ Environmentally sensitive nanomaterials, which can rapidly respond to multiple stimulations such as temperature, light, ultrasonic, magnetic field, and pH, can achieve various imaging and treatment modes, making it promising to develop an imaging‐guided combination treatment nanosystem for accurate monitoring and efficient cancer treatment.^4^

In the process of tumour treatment, imaging can provide vital information such as the size and location of the tumour and the distribution of therapeutic drugs, which is crucial for improving the accuracy of tumour treatment and reducing side effects.^5^ Nanomaterials have been widely used in traditional medical imaging fields such as magnetic resonance imaging (MRI), photoacoustic imaging (PAI), and fluorescence imaging (FLI), among others.^6^ However, a single imaging mode can only partially reflect the characteristics of different types of tumours due to the low sensitivity of MRI, poor soft tissue imaging of PAI, and poor stability of FLI.^7^ To compensate for these inherent limitations, integrating MRI, PAI, and FLI to build a multimode imaging nanosystem considering the characteristics of each imaging mode can provide accurate and rich information.

Another challenge in the current imaged‐guided combination treatment is achieving more effective cancer cell killing. NIR laser‐triggered phototheranostics have shown significant promise in cancer therapeutics due to their non‐invasive, high efficiency, and low side effects.^8^ However, this treatment strategy still has limitations in the specific tumour microenvironment.^9^ For example, after photothermal therapy (PTT), cells can acquire heat resistance leading to the recurrence of residual tumours. The tumour‐specific hypoxic microenvironment can also limit the cancer cell‐killing efficiency of photodynamic therapy (PDT) by hindering the production of ROS.^10^ Additionally, the side effects and drug resistance of chemotherapy (CMT) drugs cannot be ignored in cancer treatment.^11^ Therefore, integrating PTT/PDT/CMT triggered by NIR into a single nanosystem is expected to become an innovative strategy to overcome the shortcomings of each treatment method and achieve a synergistic treatment effect.

In this study, we reported a new all‐in‐one nano‐sphere system for cancer diagnosis and treatment. This nanosystem had excellent medical imaging capabilities, including MR, PA, and FL imaging. The combination of CMT, PTT, and PDT had dramatically improved the killing effect on cancer cells. We first used hollow mesoporous organosilica nanoparticles (HMONs)‐NH_2_ as the framework owing to its excellent loading capacity, biocompatibility, and biodegradability^12^ and grafted COOH—NGO (nano‐graphene oxide) through a covalent connection. Later, the superparamagnetic Fe_3_O_4_ nanoparticles (NPs) and hydrogen peroxide catalyst manganese oxide (MnOx) were progressively grown onto the NGO surface using a novel two‐step double redox strategy. To achieve controllable therapy, we used fatty acid (FA) as the organic phase‐change material to encapsulate the HMONs‐rNGO@Fe_3_O_4_/MnOx with drugs and photosensitizers through a simple self‐assembly process with lecithin and DSPE‐PEG5000. This excellent packaging strategy not only improved the biocompatibility of the material but also allowed it to be used for FLI.^13^ The metal oxide NPs could be used as contrast agents to enhance the visibility of specific tissues for MRI imaging. They could also increase the sensitivity and specificity of PAI. In addition, the metal oxide NPs with reduced graphene oxide could enhance photothermal efficiency in the near‐infrared (NIR) region to improve PTT. The increased temperature would also melt FA to release loaded drugs and photosensitizers for CMT and PDT. Furthermore, MnOx NPs could catalyse excessive hydrogen peroxide inside tumours into water and oxygen to sustainably produce oxygen, which enhanced tumour oxygenation in vivo and improves the productivity of ROS. All in all, the prepared HMONs‐rNGO@Fe_3_O_4_/MnOx@FA/DOX/TPP achieved triple‐modal imaging (MRI/PAI/FLI)‐guided combination tumour therapy (PTT/PDT/CMT), providing a promising nanosystem for improving the diagnosis and treatment of cancer.

## 2 RESULTS AND DISCUSSION

### 2.1 Synthesis and morphology of HMONs‐rNGO@Fe_3_O_4_ /MnOx@FA/DOX/TPP

**FIGURE 1:**
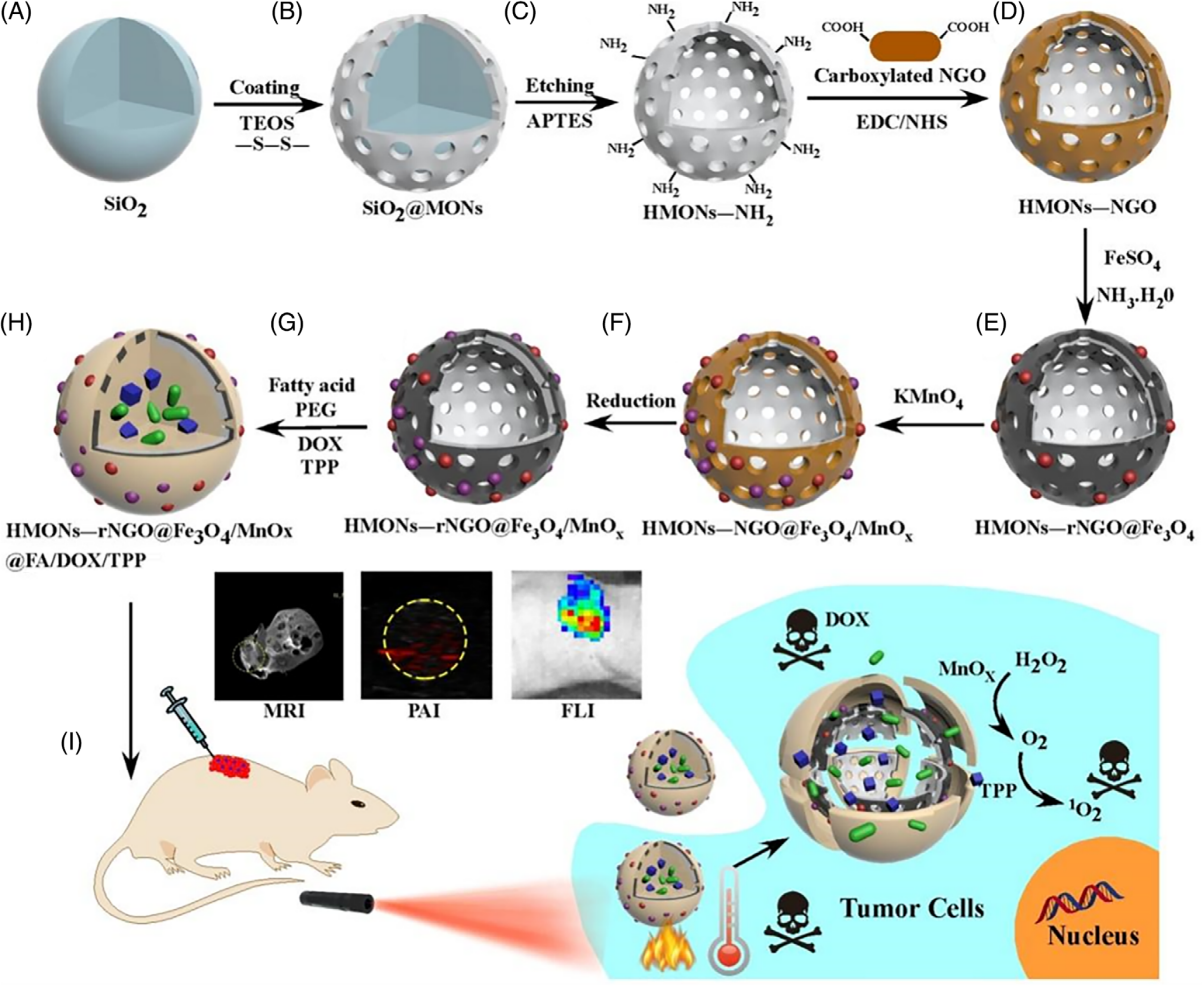
Design and fabrication of hollow mesoporous organosilica nanoparticles (HMONs)‐rNGO@Fe3O4/MnOx@FA/DOX/TPP. (A) Silicon dioxide nanoparticles (NPs). (B) Synthesis of mesoporous organosilica NPs (SiO_2_@MONs). (C) The synthesis of amino group‐modified hollow MONs (HMONs‐NH_2_). (D). Synthesis of HMONs‐graphene oxide (HMONs‐NGO) NPs through self‐assembling and condensation reaction. (E) in situ generation of superparamagnetic Fe_3_O_4_ NPs binding on the HMONs‐NGO surface. (F) In situ reduction of KMnO_4_ to produce MnOx NPs. (G) further reduction of NGO. (H) Loading DOX/TPP to HMONs‐rNGO@Fe_3_O_4_/MnOx through phase transfer and lecithin vesicle. (I) Schematic illustration showing the multimode imaging guided chemo‐photothermal‐photodynamic combination therapy triggered by near‐infrared. APTES, (3‐aminopropyl) triethoxysilane; DOX, doxorubicin; EDC, *N*‐ethyl‐*N*′‐(3‐(dimethylamino) propyl) carbodiimide; FLI, fluorescence imaging; MRI, magnetic resonance imaging; NHS, *N*‐hydroxysuccinimide; PAI, photoacoustic imaging; TEOS, tetraethylorthosilicate; TPP, tetraphenylporphyrin.

**FIGURE 2:**
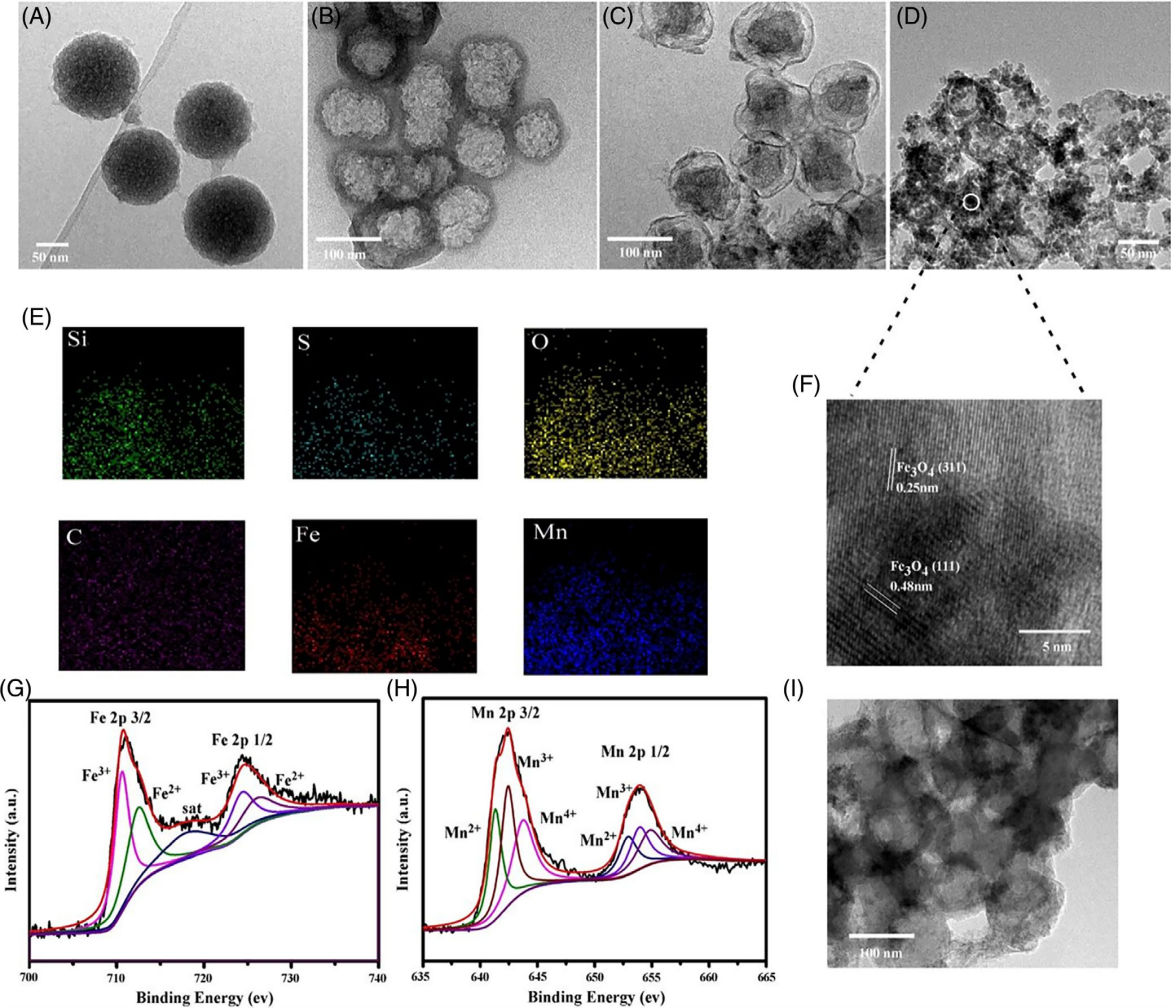
Characterization of different nanoparticles. Transmission electron microscope (TEM) images of (A) SiO_2_@MONs, (B) hollow mesoporous organosilica nanoparticles (HMONs), (C) HMONs‐NGO, and (D) HMONs‐rNGO@Fe_3_O_4_/MnOx. (E) Elemental mapping of Si, S, O, C, Fe, and Mn of HMONs‐rNGO@Fe_3_O_4_/MnOx. (F) TEM images of the HMONs‐rNGO@Fe_3_O_4_/MnOx with a high‐magnification image of crystalline. (G,H) XPS high‐resolution scans of Fe 2p, Mn 2p in HMONs‐rNGO@Fe_3_O_4_/MnOx. (I) TEM images of HMONs‐rNGO@Fe_3_O_4_/MnOx@FA/DOX/TPP.

The detailed preparation process of HMONs‐rNGO@Fe_3_O_4_/MnOx@FA/DOX/TPP was presented in Figure 1A–H. First, HMONs were synthesized with slight modifications to the previous literature.^14^ SiO_2_ NPs as core templates were synthesized via hydrolysis reaction, and the organosilica shell was formed using bis(3‐triethoxysilylpropyl) disulphide, which incorporates a disulphide bond into its framework. Following the selective etching of the core template, HMONs were produced, which were then modified with an amino group using the standard (3‐aminopropyl) triethoxysilane (APTES) method. In addition, carboxylated nano‐graphene oxide (COOH—NGO) was prepared through a modified Hummer method and the reaction with chloroacetic acid. The condensation reaction was employed to covalently graft COOH—NGO onto the surface of HMONs‐NH_2_. A novel two‐step double redox strategy was used to progressively load metal oxide NPs, including superparamagnetic iron oxide (Fe_3_O_4_) and hydrogen peroxide catalyst MnOx, onto the exfoliated NGO surface. This was achieved based on the surface redox potential changes of NGO.^15^ In this process, oxygen‐containing groups of NGO retained oxidizing potentials which could oxidize Fe^2+^ to in situ generate Fe_3_O_4_ NPs on the NGO's surface. Meanwhile, reduced NGO can be further oxidized by the introduction of KMnO_4_ leading to the generation of MnOx NPs. The NGO shell was then reduced by hydrazine to improve photothermal efficacy.^16^ To achieve controllable therapy, FA as organic phase‐change material was applied to encapsulate the HMONs‐rNGO@Fe_3_O_4_/MnOx with drug and photosensitizers. Here, we utilized lauric acid (melting point 45°C) and stearic acid (melting point 69°C) at a weight ratio of 4:1, exhibiting a melting point at 39°C.^17^ The eutectic mixture of natural FAs can maintain solid status at normal body temperature until extra heat. Figure 1I illustrated the multimode imaging guided chemo‐photothermal‐photodynamic combination therapy triggered by NIR. Upon injection into the tumour area, the HMONs‐rNGO@Fe_3_O_4_/MnOx@FA/DOX/TPP would enter tumour cells via the endocytic lysosomal pathway and accumulated in the tumour through the enhanced permeability and retention effect.^18^ MR, PA, and FL triple‐modal imaging enabled precise observation of the size and location of the tumour. This approach allowed for real‐time monitoring of treatment and minimized the risk of harming healthy tissue. Under NIR irradiation, the temperature of the tumour area increased for PTT that rNGO@Fe_3_O_4_/MnOx shell adsorbed light energy, and the FA coating was melted to release DOX and TPP for CMT and PDT. Furthermore, MnOx NPs could catalyse the conversion of hydrogen peroxide into water and oxygen, which resulted in sustainable oxygen production, enhanced tumour oxygenation in vivo, and further improved the production of singlet oxygen for PDT. Figure 2A and S2A demonstrated the structure of SiO_2_@MONs and the average diameters were around 70 nm. After etching SiO_2_@MONs in an ammonia solution, the silica core was entirely extracted, yielding HMONs (as shown in Figures 2B and S2B). Despite the etching process, HMONs still retained their uniform spherical shape and exhibited high loading capacity. The prepared COOH—NGO sheets shown in Figure S1 were grafted onto HMONs by condensation reaction. Figures 2C and S2D presented the successful surface modification in which NGO uniformly covered the whole surface of HMONs spheres. Later, Fe_3_O_4_/MnOx NPs progressively in situ grown onto NGO surface via a two‐step redox reaction shown in Figure 2D. The presence of Fe and Mn elements, uniformly covering the entire surface of the hollow sphere shown in Figure 2E, was confirmed by the element mapping. From Figure 2F, the high‐magnification transmission electron microscope (TEM) image of HMONs‐rNGO@Fe_3_O_4_/MnOx, the generation of Fe_3_O_4_ was further confirmed according to its interplanar spacing that 0.25 nm represent [311] plane and 0.48 nm represent [111] plane of Fe_3_O_4_.^19^ Based on the X‐ray photoelectron spectroscopy (XPS) result in Figure 2G,H, it could be concluded that the formation of Fe_3_O_4_ and MnOx was validated. In the Fe 2p region, there were four spectral peaks, which could be attributed to Fe^2+^ and Fe^3+^ species. The two peaks located at 725.3 and 711.9 eV corresponded to 2p1/2 and 2p3/2 of Fe^3+^ species, respectively. The other two peaks observed at a binding energy of 723.8 and 710.6 eV could be attributed to 2p1/2 and 2p3/2 of Fe^2+^ species. The presence of both Fe^2+^ and Fe^3+^ species suggests that the sample was a mixed‐valence compound. These results were also consistent with the formation of Fe_3_O_4_, which is known to contain both Fe^2+^ and Fe^3+^ ions in its crystal structure. The Mn 2p regions of the XPS spectra had Mn2p3/2 binding energy peaks located at 641.4, 642.8, and 645.9 eV, which correspond to Mn^2+^ (MnO), Mn^3+^ (Mn_2_O_3_ or Mn_3_O_4_), and Mn^4+^ (MnO_2_), respectively.^20^ Finally, the prepared HMONs‐rNGO@Fe_3_O_4_/MnOx were encapsulated by FA in Figure 2I, and the diameter of the NPs was increased. And, the drug loading contents were further determined to be 4.2% ± 0.3%. The NPs were evenly dispersed in an aqueous solution, and their average hydrodynamic diameter was determined using dynamic light scattering. Encapsulation with FA/DOX/TPP led to an increase in the diameter of the NPs to 153.5 nm, as evidenced by Figure S3. This increase in size could be due to the encapsulation of the drugs and targeting ligands onto the surface of the NPs.

### 2.2 Characterization of HMONs‐rNGO@Fe_3_O_4_ /MnOx@FA/DOX/TPP for all‐in‐one function

**FIGURE 3:**
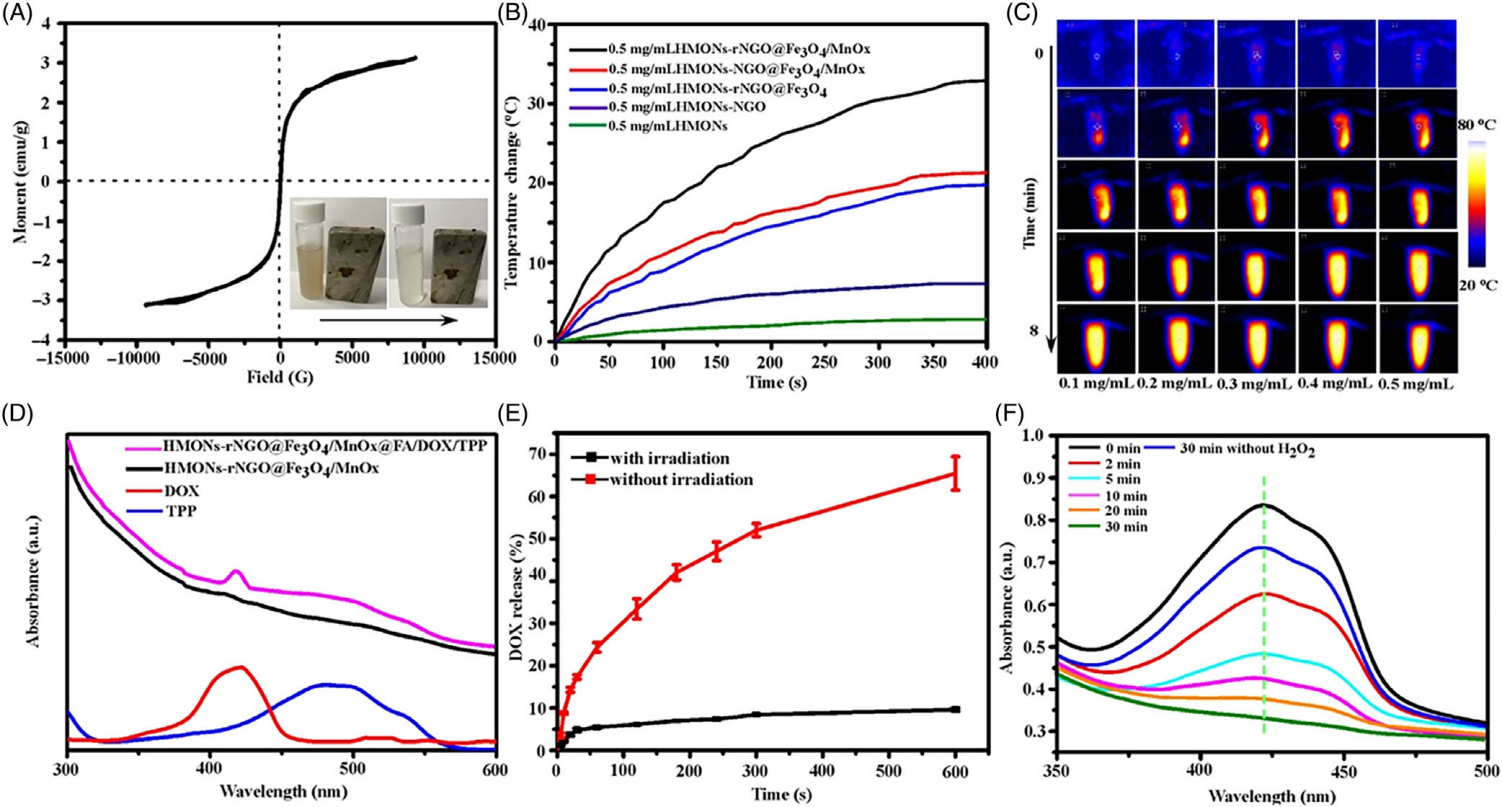
Performance of HMONs‐rNGO@Fe_3_O_4_/MnOx@FA/DOX/TPP. (A) Room temperature magnetic hysteresis loops of hollow mesoporous organosilica nanoparticles (HMONs)‐rNGO@Fe_3_O_4_/MnOx@FA/DOX/TPP and the insert is the digital photographs depicting the HMONs‐rNGO@Fe_3_O_4_/MnOx@FA/DOX/TPP solution before and after magnetic field attraction for 10 min. (B) The rate of temperature rise at the constant laser function power (880 nm, 0.8 W cm^−2^) for HMONs, HMONs‐NGO, HMONs‐rNGO@Fe_3_O_4_, HMONs‐NGO@Fe_3_O_4_/MnOx, HMONs‐rNGO@Fe_3_O_4_/MnOx solution with the same concentration (0.5 mg/mL). (C) Infrared thermal images of 0.1, 0.2, 0.3, 0.4, and 0.5 mg/mL HMONs‐rNGO@Fe_3_O_4_/MnOx solution. (D) UV–vis absorption spectra of free DOX (red), free TPP (black), (blue), HMONs‐rNGO@Fe_3_O_4_/MnOx, and HMONs‐rNGO@Fe_3_O_4_/MnOx@FA/DOX/TPP (purple) in isopropyl alcohol. (E) DOX release profiles from HMONs‐rNGO@Fe_3_O_4_/MnOx@FA/DOX/TPP NPs with or without near‐infrared (NIR) laser irradiation. (F) UV–vis absorption spectra of 1,3‐diphenylisobenzofuran with HMONs‐rNGO@Fe_3_O_4_/MnOx@FA/DOX/TPP solution under NIR laser irradiation with or without H_2_O_2_ at different time.

The saturation magnetization of HMONs‐rNGO@Fe_3_O_4_/MnOx was 3.16 emu/g (Figure 3A) and hysteresis loops with a small area represented low coercivity which was beneficial for the magnetic target. To evaluate the photothermal properties of various NPs, a solution was irradiated with an 808 nm NIR laser at a power density of 0.8 W cm^−2^. From Figure 3B, HMONs showed weak photothermal capacity and NGO coating could slightly enhance photothermal efficiency. Meanwhile, after the growth of Fe_3_O_4_ and reducing of NGO, the photothermal efficiency greatly increased which is consistent with previous reports. The high photothermal conversion performance of MnOx could further enhance the photothermal performance^21^; however, the oxidation of rNGO offset some of the enhancement. After the reduction of NGO, the temperature change of final HMONs‐rNGO@Fe_3_O_4_/MnOx could reach 33°C in 400 s which was sufficient to ablate cancer cells. The more considerable temperature variation of HMONs‐rNGO@Fe_3_O_4_/MnOx not only could enhance hyperthermia but also could quickly melt the FA dressing to trigger inclusion release. The temperature changes of HMONs‐rNGO@Fe_3_O_4_/MnOx solutions with different concentrations were confirmed by the infrared (IR) thermal images that increasing the concentration caused the rise of temperature changes. (Figure 3C). The relationship between temperature change and laser power density was then investigated. As presented in Figure S4, the temperature increased rapidly as the power density increased, and maintained a linear relationship. Meanwhile, the successful loading of DOX/TPP was proven by UV–vis spectra shown in Figure 3D, the two peaks of the purple line represented the DOX (480 nm) and TPP (419 nm) which could dissolve into isopropyl alcohol with FA from NPs. Figure 3E demonstrated that the 808 nm NIR laser could trigger DOX release. Under NIR irradiation, the release of DOX was gradually increased and reached 65% in 10 min. On the opposite, without irradiation, only 10% of DOX was released after 10 min. Accelerating the controllable release of the drug at the tumour region promotes CMT.

Besides, previous reports mentioned that inside solid tumours were a hypoxia environment leading to a significantly high level of H_2_O_2_.^22^ Thus, the ideal NPs could not only consume extra H_2_O_2_ but also generate singlet oxygen for PDT. The oxygen‐generating ability of HMONs‐rNGO@Fe_3_O_4_/MnOx in H_2_O_2_ solution was measured shown in Figure S5 that significant amounts of dissolved oxygen were produced in a short time. Furthermore, to quantitatively analyse the generation of singlet oxygen, the typical 1,3‐diphenylisobenzofuran (DPBF) agent was utilized (Figure 3F). The mechanism was that the generated singlet oxygen could oxidize DPBF to decrease its absorbance intensity in the UV–vis spectrum at the wavelength of 410 nm.^23^ In other words, the lower absorbance intensity of DPBF proved the singlet oxygen generation. Under NIR irradiation for 30 min, a significant decrease in the absorbance intensity of DPBF was observed in the presence of HMONs‐rNGO@Fe_3_O_4_/MnOx@FA/DOX/TPP, illustrating the fast production of singlet oxygen for PDT. Notably, compared with the 30 min without the H_2_O_2_ group, the slight reduction of absorbance intensity of DPBF also confirmed the catalytic action of MnOx, which consumed H_2_O_2_ and supplied O_2_ for singlet oxygen.

### 2.3 The cell‐killing effect of combination therapy triggered by NIR in vitro

**FIGURE 4:**
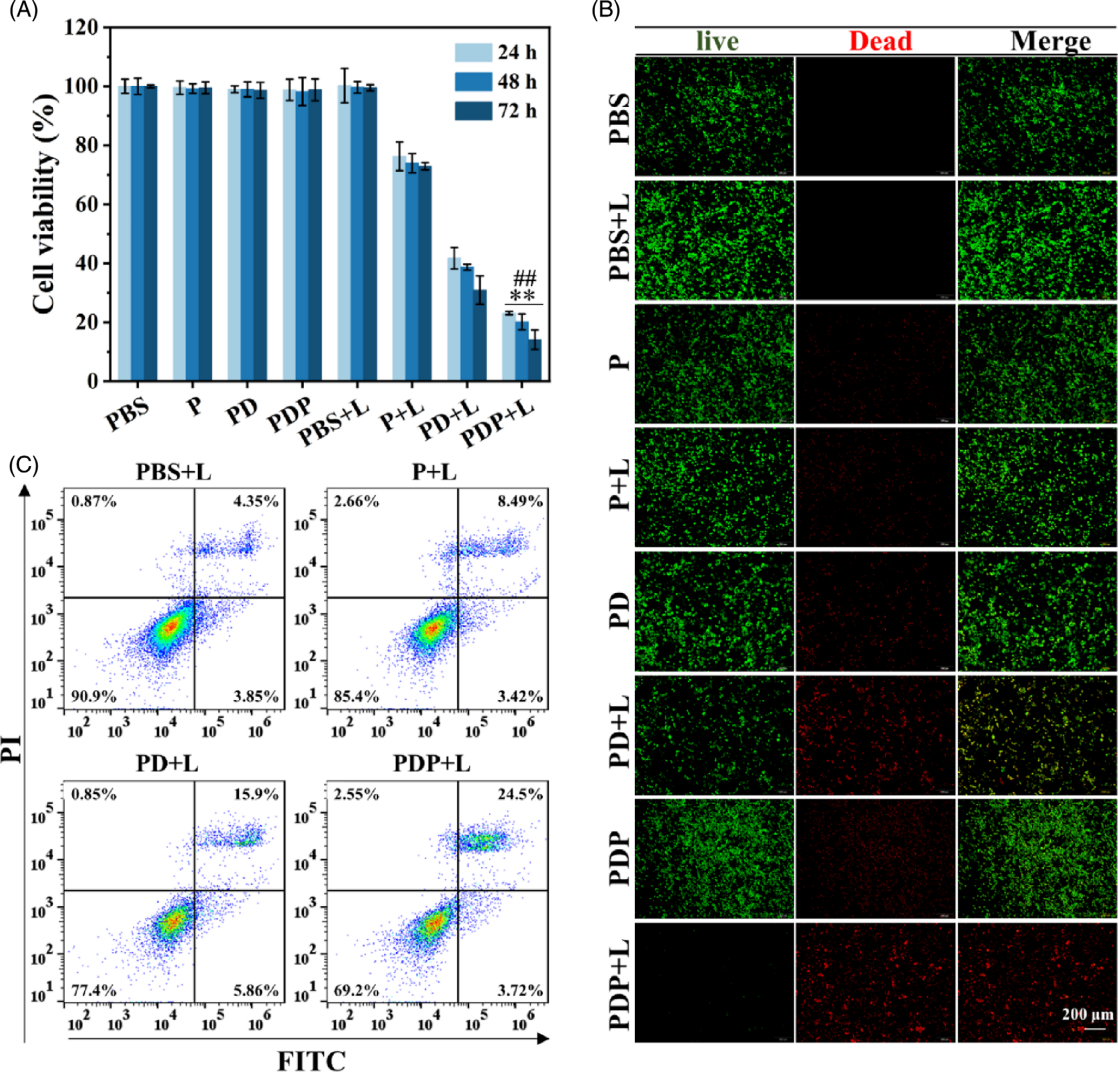
The killing effect of combination therapy of PDP in Hela cells triggered by near‐infrared. (A) Cell viability of Hela cells in different groups for 24, 48, and 72 h. (B) Live/dead staining of Hela cells in different groups at 24 h. (C) Apoptosis of Hela cells in different groups at 24 h using flow cytometry (** denotes significantly different from PBS + L, *p* < 0.01. ## denotes significantly different from PDP, *p* < 0.01).

To further detect the killing effect of combination therapy in Hela cells, six groups including HMONs‐rNGO@Fe_3_O_4_/MnOx@FA (P), P with NIR laser irradiation (P + L), HMONs‐rNGO@Fe_3_O_4_/MnOx@FA/DOX (PD), PD with NIR laser irradiation (PD + L), HMONs‐rNGO@Fe_3_O_4_/MnOx@FA/DOX/TPP (PDP), and PDP with NIR laser irradiation (PDP + L) were divided. NIR below was irradiated by a laser (808 nm, 0.8 W cm^−2^) for 10 min. The result in Figure S6 showed that the viability of Hela cells was not obviously inhibited in PDP groups with the increased concentration of PDP for 72 h, indicating the DOX and TPP were well encapsulated in PDP. To achieve the best cell‐killing effect, the concentration of PDP was chosen at 50 μg/mL in the following experiments. Moreover, the viability of Hela cells incubated with PD and PDP was significantly reduced compared with PBS groups after NIR for 10 min (Figure 4A). Importantly, higher cytotoxicity was shown in PDP, which proved that the combination treatment of PDP could kill Hela cells more effectively. This cytotoxic trend of PDP was also confirmed from the live/dead staining of Hela cells (Figure 4B). Furthermore, the flow cytometry experiment suggested that after irradiation with NIR, Hela cells incubated with PDP were significantly apoptosis for 24 h (Figure 4C). All these results indicated that the PTT/PDT/CMT combination treatment triggered by NIR of PDP was a more effective and powerful manner to kill Hela cells.

### 2.4 The biological metabolism of PDP in Hela cells

**FIGURE 5:**
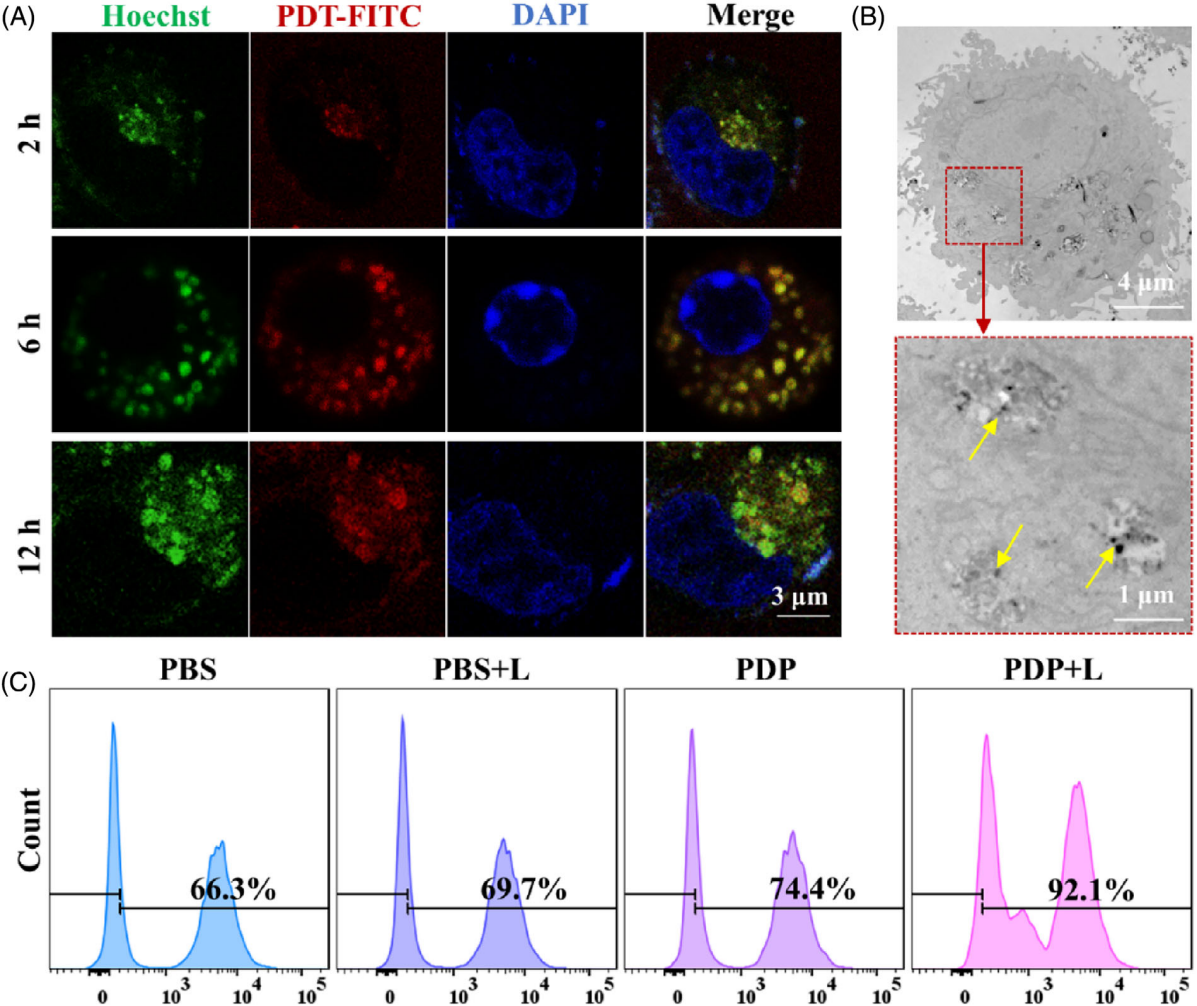
Biological metabolism and intracellular reactive oxygen species (ROS) production properties of PDP with NIR. (A) Lysosome tracer of PDP in Hela cells for 2, 6, and 12 h. (B) Transmission electron microscope images of lysosomal phagocytosis of PDP for 2 h. The yellow arrow points to PDP in the lysosome. (C) Quantitative analysis of ROS contents in Hela cells incubated with PDP for 24 h using flow cytometry.

To further investigate the metabolic pathway of PDP, scanning electron microscope (SEM) of Hela cells after incubation with PDP was performed (Figure S7). It was shown that PDP were abundantly enriched on the surface of the cell membrane and appeared to be phagocytosed after incubation for 2 h. Moreover, as shown in Figure 5A, the yellow fluorescence of colocalization in Hela cells was gradually enhanced, which revealed that PDP was phagocytic into the intracellular lysosome from 2 to 12 h. Consistent with the results of confocal FLI, the TEM images further confirmed that the PDP was accumulated in the lysosomes of Hela cells after incubation for 12 h (Figure 5B). Then, the 2,7‐dichloro‐dihydrofluorescein diacetate (DCFH‐DA) probe was applied to detect ROS contents in Hela cells to explore the effectiveness of the photocatalytic properties in PDP.^24^ As shown in Figure 5C, after irradiation with NIR, the content of ROS in Hela cells was significantly increased after incubation with PDP for 24 h, which showed that PDP could overcome tumour hypoxia by modifying the tumour microenvironment to replenish oxygenation and provided a strong capacity for generating ROS after NIR irradiation. These results indicate that PDP can be effectively swallowed into Hela cells and produce effective PDT to kill Hela cells.

### 2.5 PA imaging and MR imaging ability of PDP in vitro

**FIGURE 6:**
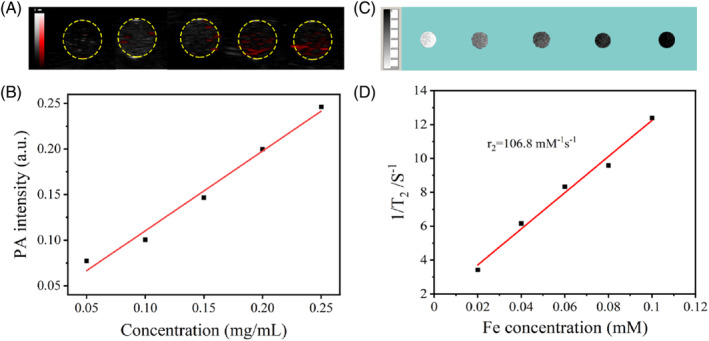
Imaging property of PDP in vitro. (A) Photoacoustic (PA) imaging of PDP at different concentrations. (B) The PA signal curve of PDP at different concentrations in (A) indicates a linear relation. (C) Magnetic resonance imaging of PDP at different concentrations. (D) The 1/T_2_ of PDP at different concentrations in (C) indicates a linear relationship, and the relaxation rate *r*_2_ = 106.8 mM^−1^ s^−1^.

The majority of the nanomaterials with photothermal properties are distributed in a way that can be detected by PA imaging, which can be used as a complement to the resolution‐deficient MRI.^25^ We first investigated the PA phantom of PDP. It was showed that the PA image of Hela cells incubated with PDP was significantly enhanced with the increase of concentration (Figure 6A,B). Moreover, the PA signals generated by PDP showed a linear relationship with their concentrations, indicating their excellent and stable PA imaging ability. In addition, it was observed from the T_2_‐weighted MRI that PDP showed a gradual decrease in the intensity of MR signal with increasing concentration of Fe (Figure 6C). By applying a linear fit to the T_2_ relaxation rate (1/T_2_) versus the concentration of Fe, the relaxation rate (*r*_2_) of PDP is about 106.8 mM^−1^ s^−1^ based on our calculations (Figure 6D). The above results imply that PDP are capable of integrating MR, PA imaging and show the potential to treat all‐in‐one multimodal imaging.

### 2.6 The multimodal imaged combination treatment of PDP in vivo

**FIGURE 7:**
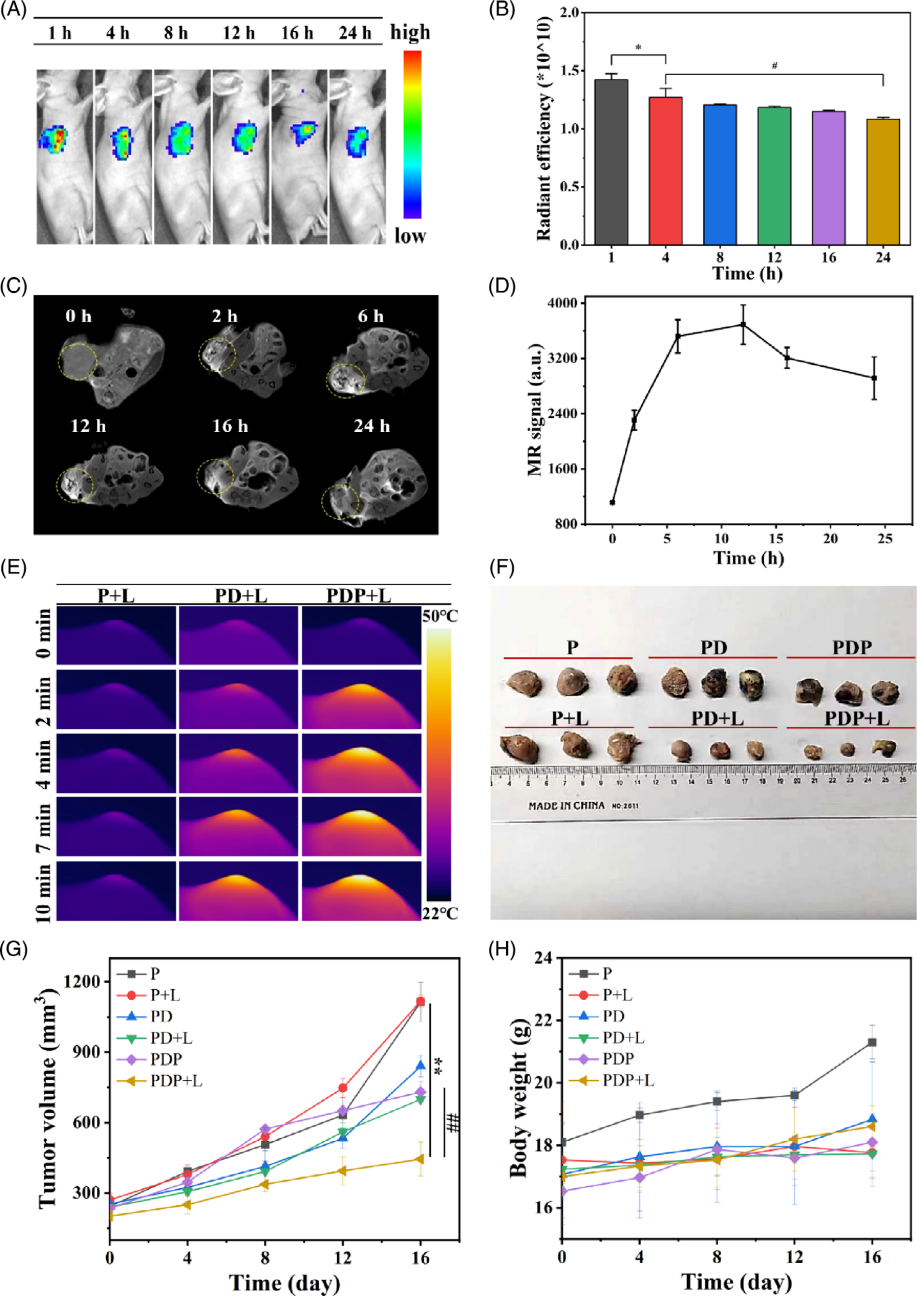
Multimodal imaged combination treatment of PDP in vivo. Fluorescence images (A) and relative quantitative analysis (B) of subcutaneous tumour‐bearing mice taken at different times after local injection of PDP. Magnetic resonance imaging (C) and relative signal intensity (D) of subcutaneous tumour‐bearing mice after PDP injection. (E) Near‐infrared (NIR) thermal images of subcutaneous tumour‐bearing mice triggered by NIR after P, PD, and PDP injection. Tumour images (F) and tumour volume (G) of different groups. (H) Body weight curves of different groups. (*and # denote difference, *p* < 0.05. ** denotes significantly different from P + L at 16 days, *p* < 0.01. ## denotes significantly different from PD + L at 16 days, *p* < 0.01).

To further confirmed the multimodal imaged combination treatment effect in vivo, subcutaneous Hela tumour‐bearing mice were first established. The FLI was used to track PDP in subcutaneous Hela tumour‐bearing mice after local injection. As shown in Figure 7A,B, the fluorescence signal in the tumour sites was apparently peaked at 1 h and still observed over 24 h after injection, indicating that PDP can be effectively accumulated in the local tumour area for FLI.^26^ Then MR images of the tumour site and the associated signal curves were acquired at different times (0, 2, 6,12, 16, and 24 h; Figure 7C). The T_1_‐weighted MR images of the tumour were observed clearly. The mean MR signal of PDP gradually increased, reaching a peak of 3690 a.u. at 12 h after injection and then decreasing to 2916 at 24 h (Figure 7D). Moreover, to detect the photothermal effect of PDP, the infrared thermography of PDP particles was studied by irradiating with NIR for 10 min after 1 h (maximum accumulation time) of injection. As shown in Figure 7E, the regional temperature of the tumours rapidly reached 43°C for 2 min and was up to as high as 50°C for 4 min in PDP groups, demonstrating that PDP provided superior photothermal performance to achieve excellent PTT. To further investigate the combination treatment effects of PDP, the subcutaneous Hela tumour‐bearing mice were randomly divided into six groups (P, P + L, PD, PD + L, PDP, and PDP + L). The tumour size and body weight were detected during the treatment to assess the treatment effects of different groups of mice. As shown in Figure 7F,G, the tumour volume was smallest in the PDP + L group, showing the best combination antitumor effects of PDP. In addition, no noticeable weight loss (Figure 7H) and tissue toxicity (Figures S8 and S9) were found in the groups, indicating excellent biosafety of PDP in vivo.

## 3 CONCLUSION

In summary, a NIR‐targeted tumour theranostic nanomedicine was developed based on a liposome‐based nanoreactor. This nanoreactor encapsulated NGO‐modified HMONs with DOX and TPP, which were further encapsulated by natural FA. The Fe_3_O_4_ and MnOx NPs were progressively grown onto the NGO surface using a novel two‐step double redox strategy. The synthesized metal oxide could enhance photothermal efficiency, improve the visibility of specific tissues for MRI, increase the sensitivity and specificity of PAI, and catalyse excessive hydrogen peroxide for PDT. After the NPs were systemically injected into tumour‐bearing mice, the tumour could be detected simultaneously by FL, PA, and MR imaging. Moreover, due to the photothermal effect of HMONs‐rNGO@Fe_3_O_4_/MnOx@FA/DOX/TPP, FA was melted under NIR irradiation to release DOX and TPP, leading to combination tumour therapy (PTT/PDT/CMT). From the in vivo results, the tumour was effectively killed by combination therapy, and no apparent side effects were observed. Overall, the NIR laser‐triggered all‐in‐one imaging‐guided therapy of the tumour presented in this work shows irreplaceable advantages in precise tumour theranostics and massive potential for clinical application.

## 4 EXPERIMENTAL SECTION

### 4.1 Characterizations

The SEM (JEOL, JAPAN) and TEM (JEOL, JAPAN) were used to observe the morphologies of the NPs. The UV–vis spectra were run on a UV‐2550 spectrophotometer (Perkin Elmer, USA) for drug loading and singlet oxygen generation. Fluorescence was measured on Varioskan LUX Multimode Microplate Reader (Thermo Fisher Scientific, USA). The room temperature hysteresis loop was performed on a vibrating sample magnetometer (Lake Shore Cryotronics, USA). The dissolved oxygen was detected using a portable dissolved oxygen meter (Leici, China).

### 4.2 Synthesis of amino group‐modified HMONs

The production of HMONs involved the following steps. First, a mixture of cetrimonium bromide (CTAB; 20 g, 10 wt%) and triethanolamine (0.8 g, 10 wt%) aqueous solutions was prepared and stirred in a water bath at 95°C. Then, tetraethylorthosilicate (TEOS; 1 mL) was added dropwise, resulting in a hydrolysis/condensation reaction that formed the SiO_2_ core within 2 h. Next, a combination of mixed silicon sources, TEOS (1 mL) and Bis (3‐ triethoxysilylproyl) disulphide (0.6 mL), was added, leading to the formation of the SiO_2_@MONs core/shell structure after 4 h. The resulting white product was collected and washed with ethanol three times. CTAB was then removed using a mixture of ethanol and concentrated HCl (37%) by extracting three times for 12 h at 80°C. The product was washed again with ethanol and dispersed into water (100 mL), followed by the addition of ammonia solution (2 mL). The etching process was carried out at 100°C for 3 h, after which the HMONs were collected by centrifugation and washed with water. To obtain HMONs modified with amino groups (HMONs‐NH_2_), the collected HMONs were dispersed in ethanol (100 mL) and APTES (1 mL) was added. The mixture was then refluxed for 12 h at 80°C.

### 4.3 Synthesis of carboxylated NGO

The preparation of the NGO involved modifying the Hummer method. Initially, 1 g of graphite powder was ground for 20 min and then added to 150 mL of concentrated sulfuric acid (98%) in an ice bath. Afterwards, 40 g of KMnO_4_ was slowly added to the mixture while stirring vigorously overnight. The flask was then transferred to an oil bath and heated to 60°C for 8 h with mechanical solid stirring. The resulting mixture was poured into 1 L of DI water, and 30% H_2_O_2_ was added dropwise with stirring until no more bubbles were produced. The GO sheets were subsequently washed multiple times with DI water. To obtain the final NGO solution, the solution was sonicated for 4 h with a tip sonicator (500 W, 10 kHz) in an ice bath. The transformation of hydroxyl, epoxide, and ester groups in the NGO into carboxylic acid (COOH) moieties was achieved through a reaction with chloroacetic acid. In detail, 4 g of KOH was dispersed in 50 mL of the NGO solution and stirred for 2 h at 100°C. Then, 1.5 g of chloroacetic acid was dissolved into the mix and stirred for 24 h. The resulting carboxylated NGO sheets were washed with DI water and ethanol multiple times and collected through centrifugation.

### 4.4 Synthesis of HMONs‐NGO

The process of anchoring the NGO onto the HMONs was carried out using an amidation reaction between the carboxyl and amino groups. Starting with 1 g of HMONs‐NH_2_, the first step was to disperse it in a 100 mL MES buffer solution (0.1 moL/L, pH = 5.5). Then, *N*‐ethyl‐*N*′‐(3‐(dimethylamino) propyl) carbodiimide (20 mg) and *N*‐hydroxysuccinimide (20 mg) were added. Next, carboxylated NGO (30 mg) was slowly added to the mixture and stirred at room temperature for 12 h. Afterwards, the resulting mixture was centrifuged and washed with DI water until the supernatant became clear.

### 4.5 Synthesis of HMONs‐rNGO@Fe_3_O_4_ /MnOx

To start, HMONs‐NGO was dispersed in a 1 mg/mL aqueous solution using ultrasonic treatment. Fe_3_O_4_ was then loaded by adding 5 mL of ammonia solution (25%) to a GO solution (1 mg/mL, 100 mL) under magnetic stirring at room temperature. Next, a freshly prepared solution of FeSO_4_·7H_2_O (0.5 M, 5 mL) was slowly added to the HMONs‐NGO solution, and the reaction was allowed to proceed for 1 h. The resulting product was collected via centrifugation, washed three times with water, and redispersed in a 100 mL aqueous solution using ultrasonic treatment. Then, a freshly prepared solution of KMnO_4_ (0.5 M, 5 mL) was gradually added to the HMONs‐rNGO@Fe_3_O_4_ dispersion under magnetic stirring in the dark for 5 h at room temperature. The resulting HMONs‐NGO@Fe_3_O_4_/MnOx product was collected via centrifugation, washed six times with water to remove unreacted MnO_4_^−^, and then dispersed in an aqueous solution. Finally, 0.5 mL of hydrazine was added to a 50 mL HMONs‐NGO@Fe_3_O_4_/MnOx solution and the mixture was heated to 90°C for 2 h. The prepared HMONs‐rNGO@Fe_3_O_4_/MnOx was collected via centrifugation and washed with water three times.

### 4.6 Synthesis of HMONs‐rNGO@Fe_3_O_4_ /MnOx@FA/DOX/TPP

Initially, 40 mg of lauric acid and 10 mg of stearic acid were dissolved in 4 mL of methanol, followed by the addition of HMONs‐rNGO@Fe_3_O_4_/MnOx and sonication until the concentration reached 5 mg/mL. Lecithin and DSPE‐PEG5000 were dissolved in a 4% aqueous ethanol solution at a concentration of 1 mg/mL and heated to 60°C. The FA/HMONs‐rNGO@Fe_3_O_4_/MnOx solution was mixed with 400 μL of 2.5 mg/mL DOX in DMSO and 100 μL of 2.5 mg/mL TPP in DMSO, then dropwise added to the preheated phospholipid solution while sonicated strongly for 20 min, until the solution cooled down to room temperature. The final solution was filtered through a surfactant‐free cellulose acetate membrane, and unencapsulated molecules and organic solvents were removed using centrifugation. The resultant NPs were washed three times with water and suspended in water for further use.

### 4.7 The photothermal effect of HMONs‐rNGO based nanosystem

Different concentrations of HMONs, HMONs‐NGO, HMONs‐rNGO@Fe_3_O_4_, HMONs‐NGO@Fe_3_O_4_/MnOx, and HMONs‐rNGO@Fe_3_O_4_/MnOx solutions were subjected to an 808‐nm NIR laser at varying power densities, and the resulting temperature was measured using a thermal imager (UTi165K).

### 4.8 Drug loading content and NIR‐triggered drug release

The drug loading content was determined as the percentage of the weight of the drug‐loaded to the weight of the NPs by multiplying with 100%. To evaluate the DOX release profile under NIR irradiation, a 2 mL aqueous solution of HMONs‐rNGO@Fe_3_O_4_/MnOx@FA/DOX/TPP with a concentration of 0.5 mg/mL was exposed by a laser (808 nm, 0.8 W cm^−2^). At specified time intervals, a 10 μL sample of the irradiated solution was collected for fluorescence measurements using a microplate reader (*E*_*x*_ = 488 nm, *E*_*m*_ = 590 nm). The DOX release rate was determined by (*I*_t_ − *I*_0_)/(*I*_max_ − I0) × 100%, where *I*_t_ is the fluorescence intensity of the solution at the given time point, *I*_0_ is the initial fluorescence intensity of the solution before the test, and *I*_max_ is the fluorescence intensity of DOX at the initial concentration.

### 4.9 ROS measurements in vitro

To monitor the singlet oxygen produced by HMONs‐rNGO@Fe_3_O_4_/MnOx@FA/DOX/TPP, DPBF was utilized as a probe. The absorption peak of DPBF in the range of 350–500 nm was measured at different time points during irradiation. Prior to the test, all solutions were purged with nitrogen gas for 1 h to remove dissolved oxygen.

### 4.10 Cell culture

According to the instruments, Hela cells obtained from American Type Culture Collection (ATCC) were cultured in Dulbecco's Modified Eagle Medium containing 10% fetal bovine serum, (Hyclone, Houston, Texas), 100 U mL^−1^ of penicillin and 100 μg/mL of streptomycin (denoted as basal growth media) at 37°C in a humidified incubator with 5% CO_2_.

### 4.11 Cell viability assay and apoptosis detection

For live/dead staining, after being seeded in confocal microscopy dishes, Hela cells (5 × 10^5^ cells per well) were cultured for 24 h and then treated with different groups (PBS, PBS + L, P, P + L, PD, PD + L, PDP, and PDP + L) for 24 h. L indicates NIR irradiated by a laser (808 nm, 0.8 W cm^−2^) for 10 min. The medium was removed, and the calcein/propidium iodide (PI) Cell Viability/Cytotoxicity Assay Kit (Beyotime, Jiangsu, China) was applied. The 1 mL of dye solution containing 1 μL of Calcein AM and PI was added to each confocal dish. After incubation for 30 min, Hela cells were observed using a confocal laser scanning microscope (Leica, Wetzlar, Germany).

For the cell viability, Hela cells (1 × 10^4^ cells per well) were plated in a 96‐well plate and cultured with different treatments (PBS, PBS + L, P, P + L, PD, PD + L, PDP, and PDP + L) at 37°C for 24 h. After incubation for 24, 48, and 72 h, cells were incubated in 10% CCK‐8 reagent (Dojindo, Kumamoto, Japan) for 2 h at 37°C. The absorbance was then measured via a microplate reader at 450 nm (Biotek Synergy Neo2, Winooski, Vermont).

For the cell apoptosis, after seeded for 24 h, Hela cells (1 × 10^6^ cells/well) were treated with different groups (PBS, PBS + L, P, P + L, PD, PD + L, PDP, and PDP + L) for 24 h. The cells were digested, centrifuged, washed, and then stained with annexin 5‐fluorescein isothiocyanate (V‐FITC) and propidium iodide (PI) in accordance with the manufacturer's instructions (Solarbio, Beijing, China). Hela cells were analysed using a FACScalibur instrument (BD Biosciences, San Jose, Californina).

### 4.12 SEM and TEM images of Hela cells

For SEM images, Hela cells (1 × 10^5^ cells per well) were seeded in 6‐well plates and incubated with PDP (50 μg/mL) for 2 h. Then, the cells were fixed with glutaraldehyde solution (2.5%; Solarbio, Beijing, China) at 4°C for 1 h. After washing with PBS, the samples were dehydrated in ethanol solutions at different concentrations (30%, 50%, 70%, 90%, and 100%, vol/vol^−1^) for 15 min. Finally, the samples were dropped on the slides, air dried, and observed using SEM (S‐4800, HITACHI).

For TEM images, Hela cells (1 × 10^6^ cells per well) were seeded in 6‐well plates and cultured with PDP (50 μg/mL) for 12 h. Then, the treated cells were fixed with glutaraldehyde solution (2.5%; Solarbio, Beijing, China) at 4°C overnight. Finally, the samples were then sliced into thin chips and observed using TEM (Hitachi, Tokyo, Japan).

### 4.13 ROS detection in Hela cells

Hela cells (1 × 10^5^ cells per well) were seeded in 6‐well plates and cultured with different treatments (PBS, PDP, PDP + L) for 24 h. 5 μM DCFH‐DA (MCE, Shanghai, China) probe was added into each well for 30 min at 37°C. Then Hela cells were suspended and washed with PBS. Then, the content of ROS was further analysed by a FACScalibur instrument (BD Biosciences, San Jose, California).

### 4.14 PA and MR imaging of PDP in vitro

PDP at different concentrations were imaged after being incubated with Hela cells (1 × 10^5^ cells seeded in 6‐well plates) for 24 h. PA imaging of PDP in a single EP tube was captured by a PA imaging system (Fujifilm Visual Sonics Vevo LAZR, Japan) at different concentrations (0.05, 0.1, 0.15, 0.2, and 0.25 mg/mL) with an excitation wavelength at 808 nm. For T_2_‐weighted MRI evaluation, PDP at elevated Fe concentrations (0.02, 0.04, 0.06, 0.08, and 0.1 mM) were measured by an MR analysis system (MesoMR23‐060‐I, Suzhou, China). Experimental conditions: For T_2_‐weighted images: recycle time and echo delay time were 2000 and 80 ms, respectively.

### 4.15 Multimodal image guided combination therapy in vivo

To establish the tumour model, male Balb/c nude mice (4 weeks) were purchased from Beijing Vital River Laboratory Animal Technology Co., Ltd (Beijing, China). The animal studies were approved by Peking University Biomedical Ethics Committee. Hela cells (5 × 10^6^) suspended in 30 μL PBS were injected subcutaneously into the back of mice. Subcutaneous Hela tumour‐bearing mice (when the tumour volume reached approximately 60 mm^3^) were randomly divided into six groups (P, P + L, PD, PD + L, PDP, and PDP + L). The mice were locally injected with particles at 100 μg/mL, and L was NIR irradiated by a laser (808 nm, 0.8 W cm^−2^) for 10 min every 3 days. The tumour volume and body weight were recorded every 2 days within 16 days. The mice were dissected after therapy, and the final tumour and major organs were further studied by hematoxylin and eosin (H&E) staining.

An infrared thermal imaging camera (E50, FLIR) was used to acquire real‐time NIR imaging for 0, 2, 4, 7, and 10 min after injection. The IVIS Lumina K in vivo FLI system (PerkinElmer) was utilized to capture the fluorescence images and signals of PDP at 1, 4, 8, 12, 16, and 24 h after injection. Additionally, MRI was performed using a 3.0 T clinical MRI scanner (GE Healthcare) equipped with a small animal imaging loop.

### 4.16 Statistical analysis

All experiments data were analysed using GraphPad Prism 8.0 software (GraphPad Software Inc.), evaluated as mean ± standard deviation based on at least three tests, and contrasted via one‐way analysis of variance (ANOVA).

## CONFLICT OF INTEREST STATEMENT

The authors have no conflicts of interest to declare.

## Supporting information


**FIGURE S1:** Transmission electron microscope images of carboxylated nano‐graphene oxide (COOH—NGO)
**FIGURE S2:** Scanning electron microscope images of different nanoparticles.
**FIGURE S3:** Size distribution of HMONs, HMONs‐NGO@Fe3O4/MnOx, and HMONs‐rNGO@Fe3O4/MnOx@FA/DOX/TPP nanoparticles determined by dynamic light scattering.
**FIGURE S4:** Temperature changes of HMONs‐rNGO@Fe3O4/MnOx aqueous solutions (0.5 mg/mL) with time under laser irradiation of different powers.
**FIGURE S5:** In vitro evaluation of oxygen generation of HMONs‐rNGO@Fe3O4/MnOx in H_2_O_2_ solution (10–4 M) under pH = 7.
**FIGURE S6:** Cell viability of Hela cells treated with different concentrations of PDP at 24, 48, and 72 h.
**FIGURE S7:** Scanning electron microscope images of Hela cells incubated with PDP for 2 h. The red particles represent PDP.
**FIGURE S8:** H&E staining of different tissues in subcutaneous Hela tumour‐bearing mice after treating with PDP for 16 days.
**FIGURE S9:** Fluorescence images of subcutaneous tumour‐bearing mice taken after local injection of PDP at 36 and 48 h. The decreased fluorescence trend with the increase of injection time indicates that PDP has excellent metabolic activity.

## Data Availability

The data that support the findings of this study are available from the corresponding author upon reasonable request.
