# Supplementary material for: Hollow mesoporous organosilica nanoparticles reduced graphene oxide based nanosystem for multimodal image‐guided photothermal/photodynamic/chemo combinational therapy triggered by near‐infrared
Source: Cell Prolif. 2023 Mar 20;56(10):e13443. doi: 10.1111/cpr.13443 (PMC10542620; doi:10.1111/cpr.13443)
Supplement: Supplementary file 1 — FIGURE S1: Transmission electron microscope images of carboxylated nano‐graphene oxide (COOH—NGO) FIGURE S2: Scanning electron microscope images of different nanoparticles. FIGURE S3: Size distribution of HMONs, HMONs‐NGO@Fe3O4/MnOx, and HMONs‐rNGO@Fe3O4/MnOx@FA/DOX/TPP nanoparticles determined by dynamic light scattering. FIGURE S4: Temperature changes of HMONs‐rNGO@Fe3O4/MnOx aqueous solutions (0.5 mg/mL) with time under laser irradiation of different powers. FIGURE S5: In vitro evaluation of oxygen generation of HMONs‐rNGO@Fe3O4/MnOx in H2O2 solution (10–4 M) under pH = 7. FIGURE S6: Cell viability of Hela cells treated with different concentrations of PDP at 24, 48, and 72 h. FIGURE S7: Scanning electron microscope images of Hela cells incubated with PDP for 2 h. The red particles represent PDP. FIGURE S8: H&E staining of different tissues in subcutaneous Hela tumour‐bearing mice after treating with PDP for 16 days. FIGURE S9: Fluorescence images of subcutaneous tumour‐bearing mice taken after local injection of PDP at 36 and 48 h. The decreased fluorescence trend with the increase of injection time indicates that PDP has excellent metabolic activity. [file CPR-56-e13443-s001.docx]

Supporting Information

**HMONs-rNGO based nanosystem for Multimodal image-guided Photothermal/Photodynamic/Chemo combination therapy** **triggered by NIR**

*Chenguang Zhang, Yuting Cai, Pengrui Dang, Jiechen Wang, Lu Wang, Jiayun Xu, Yuhan Wu, Wenwen Liu*, Lili Chen*, Zhengtang Luo*, feilong Deng**


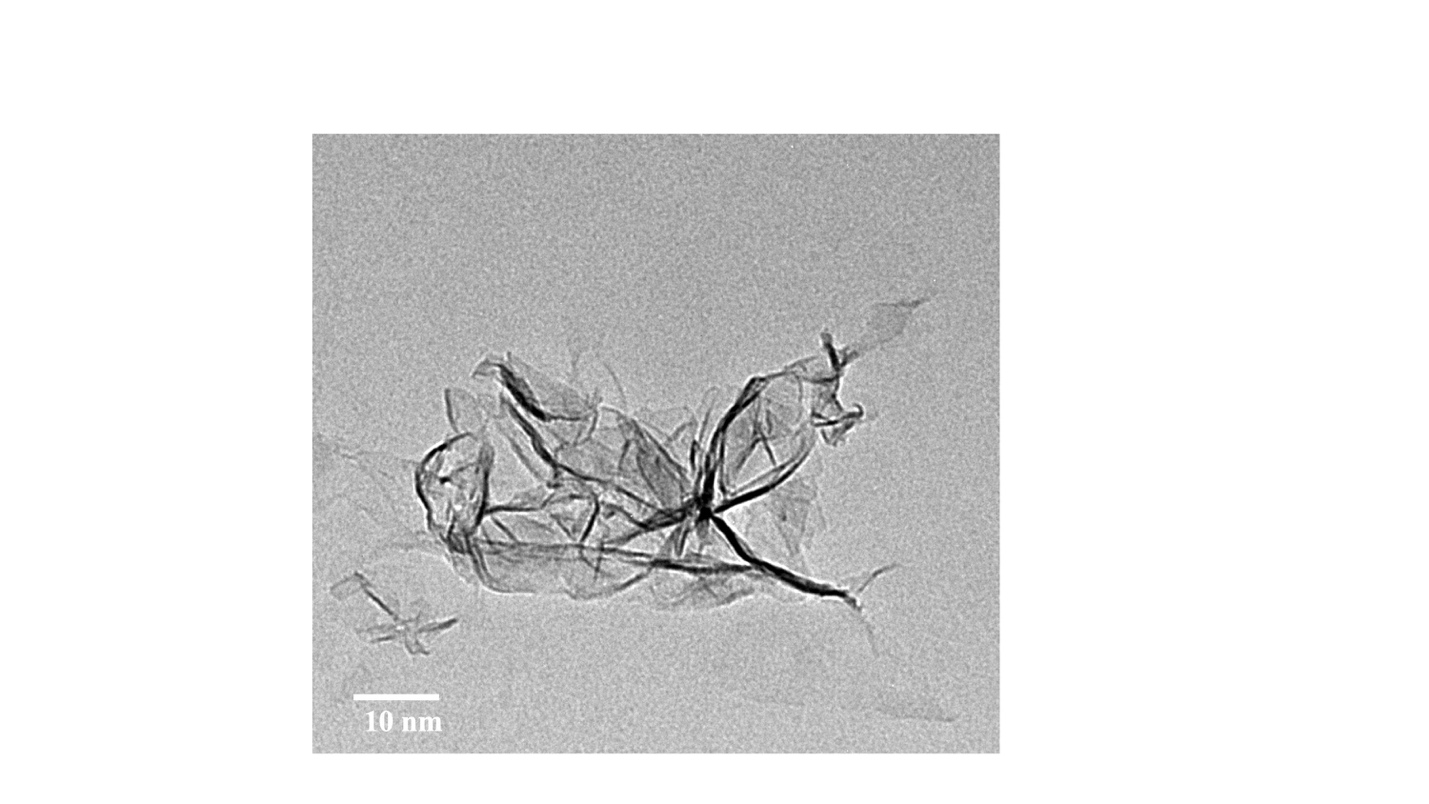


**Figure S1.** TEM images of carboxylated nano graphene oxide (COOH-NGO)


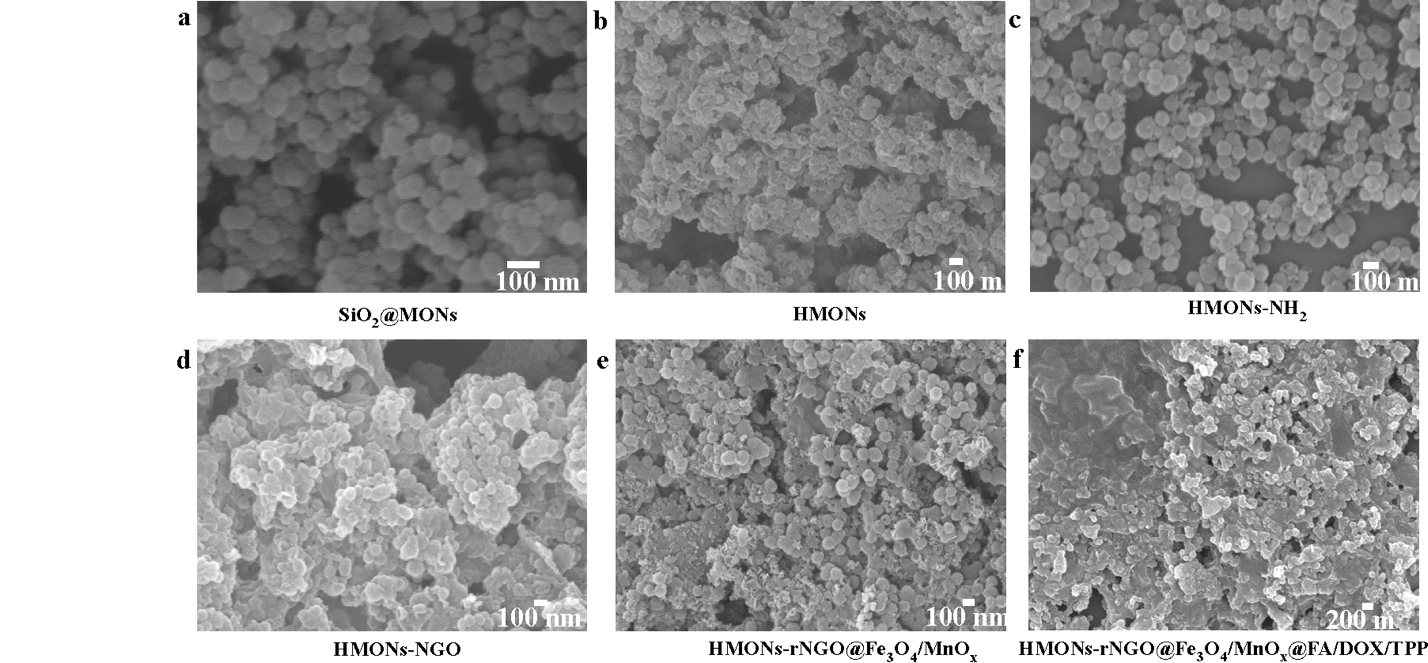


**Figure S2.** SEM images of different nanoparticles.


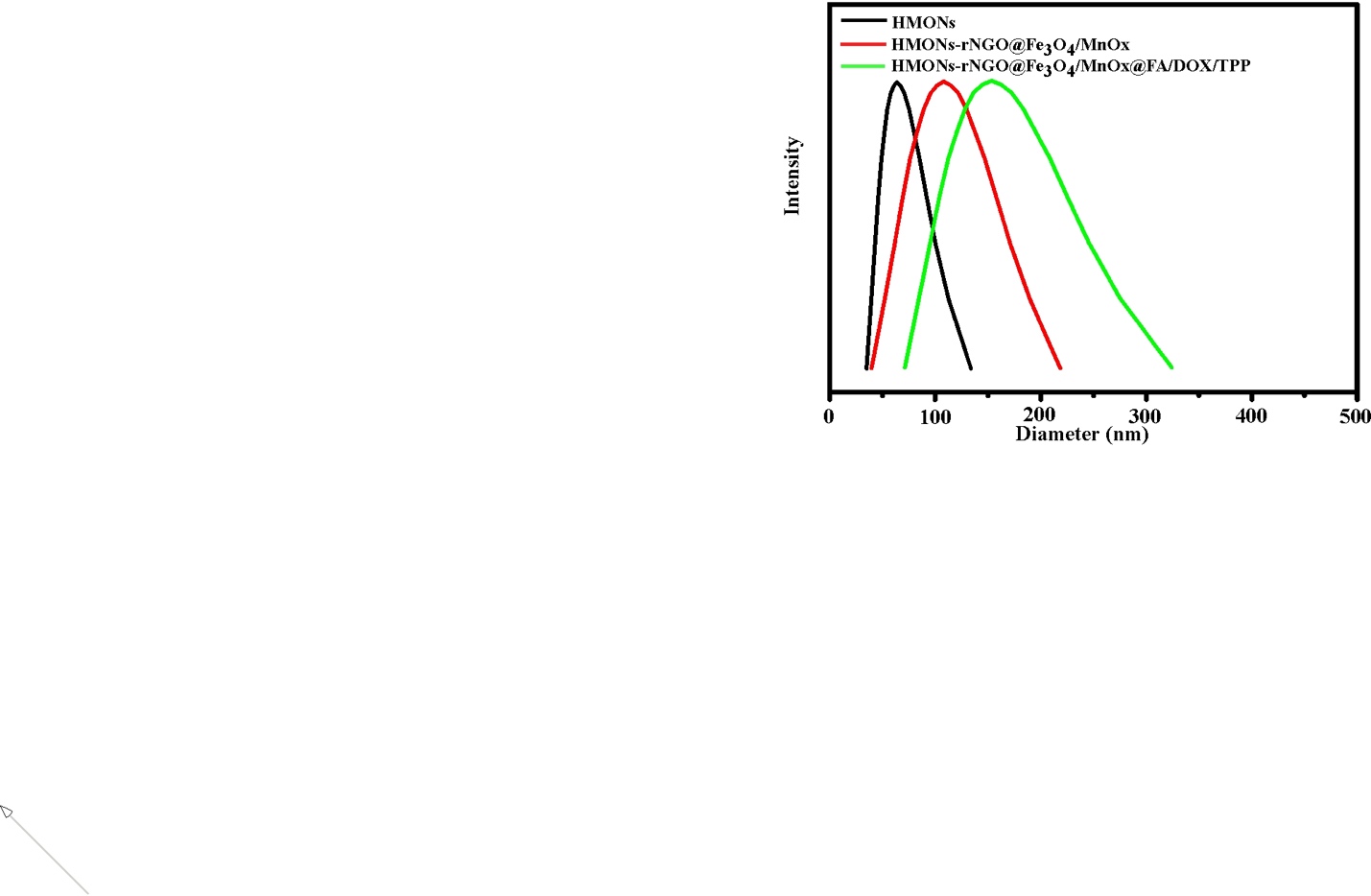


**Figure S3.** Size distribution of HMONs, HMONs-NGO@Fe_3_O_4_/MnO_X_ and HMONs-rNGO@Fe_3_O_4_/MnO_X_@FA/DOX/TPP nanoparticles determined by DLS.


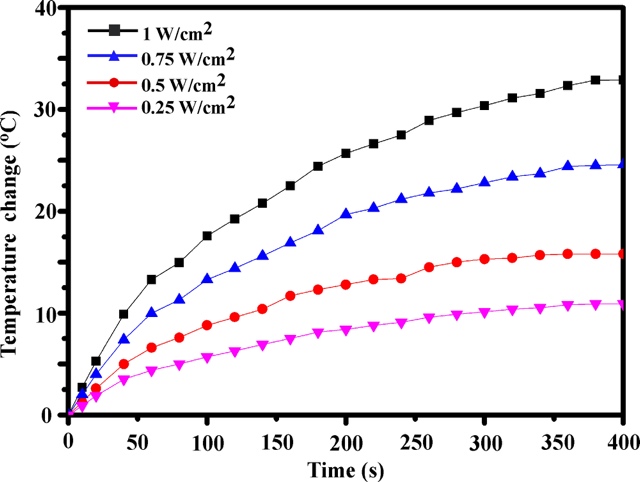


**Figure S4.** Temperature changes of HMONs-rNGO@Fe_3_O_4_/MnO_X_ aqueous solutions (0.5 mg/mL) with time under laser irradiation of different powers.


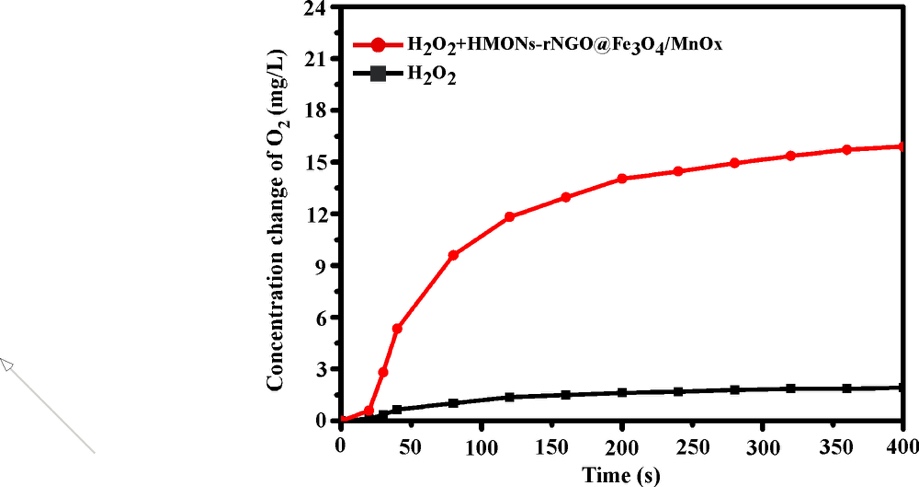


**Figure S5.** *In vitro* evaluation of oxygen generation of HMONs-rNGO@Fe_3_O_4_/MnO_X_ in H_2_O_2_ solution (10^−4^ M) under pH = 7.

**Figure S6.** Cell viability of Hela cells treated with different concentrations of PDP at 24, 48, 72 h.

**Figure S7.** SEM images of Hela cells incubated with PDP for 2 h. The red particles represent PDP.

**Figure S8.** H&E staining of different tissues in subcutaneous Hela tumor-bearing mice after treating with PDP for 16 days.


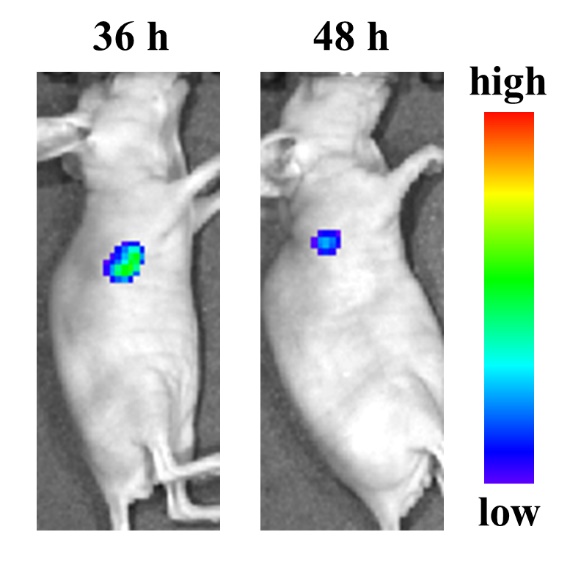


**Figure S9.** Fluorescence images of subcutaneous tumor-bearing mice taken after local injection of PDP at 36 and 48 h. The decreased fluorescence trend with the increase of injection time indicates that PDP has excellent metabolic activity.
